# Advanced Porous Gold-PANI Micro-Electrodes for High-Performance On-Chip Micro-Supercapacitors

**DOI:** 10.1021/acs.nanolett.4c03194

**Published:** 2024-08-26

**Authors:** Nibagani Naresh, Yijia Zhu, Yujia Fan, Jingli Luo, Tianlei Wang, Ivan P. Parkin, Buddha Deka Boruah

**Affiliations:** † Institute for Materials Discovery, University College London, London WC1E 7JE, United Kingdom; ‡ Department of Chemistry, University College London, London WC1H 0AJ, U.K.

**Keywords:** Micro-supercapacitors, porous interdigitated electrodes, effective material loading, rapid ions diffusion

## Abstract

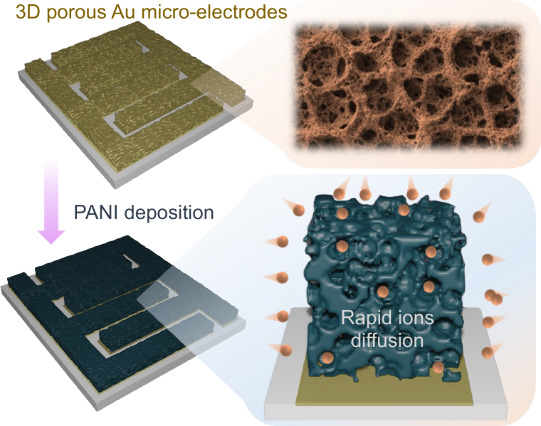

The downsizing of microscale energy storage devices is crucial for powering modern on-chip technologies by miniaturizing electronic components. Developing high-performance microscale energy devices, such as micro-supercapacitors, is essential through processing smart electrodes for on-chip structures. In this context, we introduce porous gold (Au) interdigitated electrodes (IDEs) as current collectors for micro-supercapacitors, using polyaniline as the active material. These porous Au IDE-based symmetric micro-supercapacitors (P-SMSCs) show a remarkable enhancement in charge storage performance, with a 187% increase in areal capacitance at 2.5 mA compared to conventional flat Au IDE-based devices, despite identical active material loading times. Our P-SMSCs achieve an areal capacitance of 60 mF/cm^2^, a peak areal energy density of 5.44 μWh/cm^2^, and an areal power of 2778 μW/cm^2^, surpassing most reported SMSCs. This study advances high-performance SMSCs by developing highly porous microscale planar current collectors, optimizing microelectrode use, and maximizing capacity within a compact footprint.

On-chip microelectronic devices designed for wearables and implants have made remarkable progress and are on the verge of becoming integral parts of our daily lives. These tiny devices excel in intricate tasks like data processing and wireless signal transmission within a space smaller than half a square centimeter, holding immense potential in fields such as health monitoring, medical diagnosis, and disease treatment.^1−3^ To power these devices, a crucial component is the compatible on-chip energy storage unit, which includes microbatteries and micro-supercapacitors. Microbatteries offer high energy density, while micro-supercapacitors provide high power along with rapid charging capabilities and long-cycling stability.^4−6^ However, despite their high-power density, the energy density of micro-supercapacitors is limited and needs improvement before they can be fully realized in on-chip energy storage domains. In terms of device geometries, the conventional sandwich type of micro-supercapacitors—resembling layered sandwiches with positive and negative electrodes separated by separators—features simple electrode processing.^7^ However, integrating such devices with on-chip systems to power them seamlessly poses challenges. In contrast, planar interdigitated electrode (IDE) designs offer the advantages of direct printing with devices to create a *system-on-chip* (SOC). These designs also provide better control over critical battery parameters, including internal resistance and ionic diffusion distance, all without the need for a separator.^8^ This practical solution reduces the size of micro-supercapacitors. Therefore, it is not surprising that the development of high-performance planar micro-supercapacitors has garnered significant research interest.

The effective loading of active materials onto the miniature IDEs of micro-supercapacitors plays a crucial role in overall device performance, especially given the restricted device footprint. Consequently, various advanced techniques—including 3D printing,^9^ laser scribing,^10^ screen printing,^11^ mask-assisted spray processing,^12^ and electrodeposition^13^—have been applied to load active materials onto IDEs current collectors or to directly pattern materials to serve as both IDEs and charge storage materials. Each technique offers different advantages; however, among them, electrodeposition is widely explored for loading active materials onto planar IDEs. However, processing thick, high-capacitance electrode materials through electrodeposition is significantly challenging due to their conductivity issues or the tendency to peel off from the IDEs due to insufficient adhesion with the IDEs current collectors. Therefore, intelligent IDE current collector designs are required for the effective loading of active materials to significantly boost charge storage performance within restricted device footprints. This research presents advanced IDE current collector designs to address the aforementioned challenges by exploring highly porous gold (Au) IDEs patterned on flat Au IDEs current collectors to load active supercapacitor materials. Polyaniline (PANI) is chosen as the active material for its electrical double layer and pseudocapacitive charge storage responses while maintaining material safety.^14,15^ Impressively, the PANI loaded onto porous Au IDEs based symmetric micro-supercapacitors (named P-SMSC) demonstrates significantly better charge storage performance than PANI loaded onto flat Au IDEs (SMSC). Our P-SMSC even surpasses the performance of most reported micro-supercapacitors, including those with symmetric and asymmetric electrode designs. Post-mortem analysis after long-term cycling reveals that the electrode materials and IDEs maintain identical states and morphologies, supporting the stability of our approach. We strongly believe this study contributes significantly to the development of advanced microelectrodes, thereby advancing high-performance microscale energy storage devices.

The development of porous Au IDEs is illustrated in Figure 1a. The process begins with a ceramic substrate to minimize current leakage, followed by the patterning of Au IDEs with 200 μm wide fingers and 200 μm gaps. The active area, including the gaps between the electrodes, is approximately 0.36 cm^2^. For a fair comparison, we electrodeposit PANI onto flat Au IDEs, termed SMSC (Figure 1b). Porous Au electrodes are deposited onto flat Au IDEs (Figure 1c) using an electrodeposition technique (see experimental section), and then PANI is loaded to fabricate P-SMSC (Figure 1d). The electrodeposition times of PANI on both types of electrodes were kept the same. The electrodeposition time was varied to load different masses of material and compare the results (details in the experimental section). It is noted that the porous IDEs facilitate rapid diffusion of electrolyte ions for effective interaction during the charge storage process leading to improved charge storage performance (see further). Incorporating porous Au electrodes enhances rapid electron transport through the electrodes (Figure 1e) and ensures efficient loading of active materials onto the porous networks. Digital images of the devices, including (i) Au IDEs, (ii) porous Au IDEs, (iii) SMSC, and (iv) P-SMSC, are shown in Figure 1f. The 2D and 3D representations of SMSC (Figure 1g) and P-SMSC (Figure 1h) obtained by using a profilometer technique highlight the successful loading of electrode materials onto both flat and porous Au IDEs without issues such as short circuits. Moreover, the electrode thicknesses calculated from the profilometer height profiles are measured to be 4 μm for SMSC and 16 μm for P-SMSC, excluding the thickness of the flat Au IDEs, which is around 4 μm (Figure S1). This indicates that the porous Au IDEs allow more effective deposition of PANI compared to flat Au IDEs, likely due to the geometry of the porous electrodes, which facilitates more material plating, even under the same deposition conditions for achieving higher charge storage performance (details in the experimental section).

**Figure 1:**
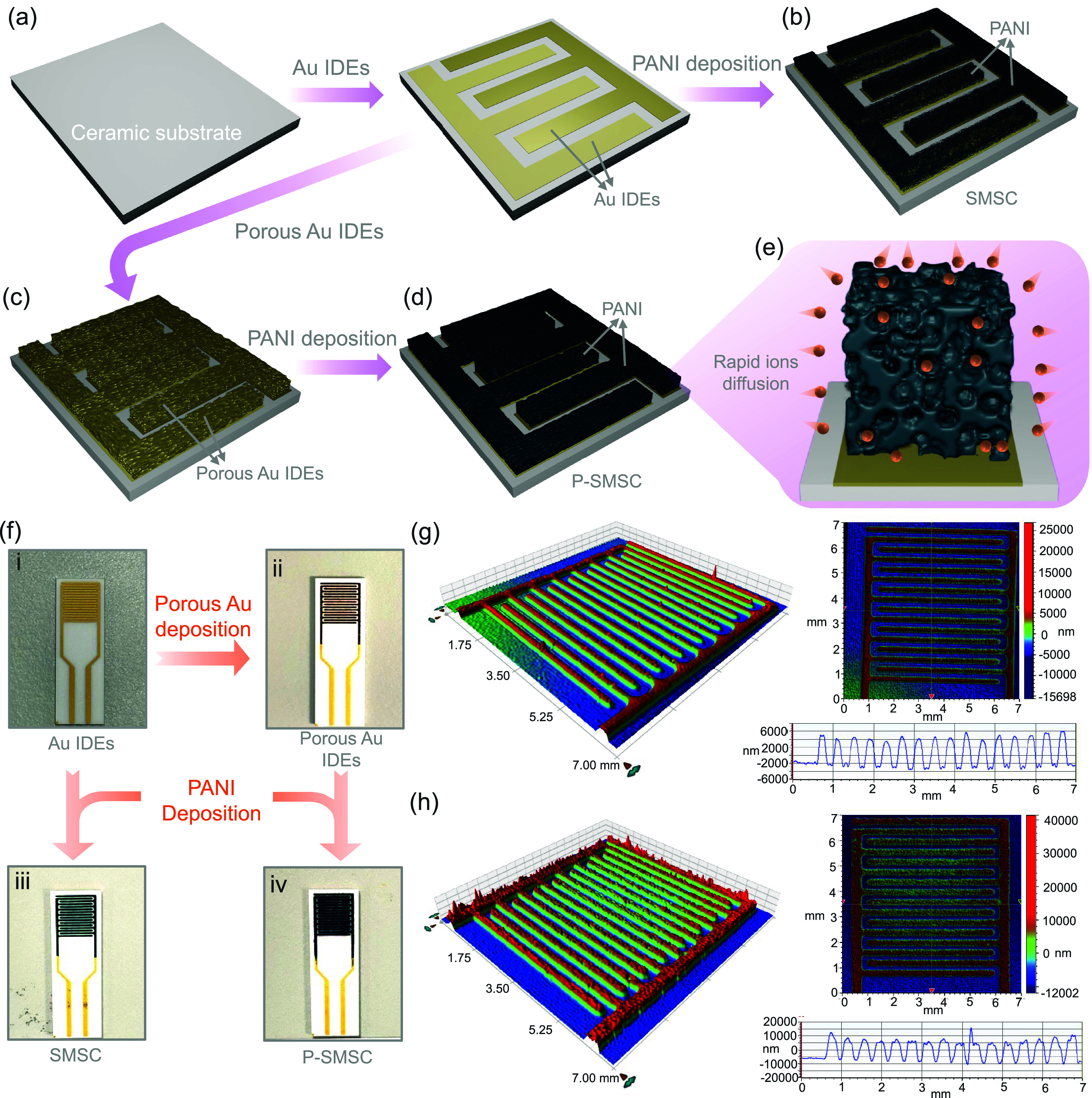
Sequential stages in the fabrication of (a) flat Au IDEs, (b) SMSC, (c) porous Au IDEs, and (d) P-SMSC. (e) Schematic illustration showing rapid 3D diffusion of electrolyte ions for efficient charge storage in P-SMSC electrodes. (f) Digital images of the (i) Au IDEs, (ii) porous Au IDEs, (iii) SMSC, and (iv) P-SMSC, where the electrodeposition of PANI was conducted for 20 s. 2D and 3D profilometer mappings of the (g) SMSC and (h) P-SMSC, showing the respective average electrode height profiles with thicknesses of 4 μm for SMSC and 16 μm for P-SMSC (excluding Au current collectors thickness).

Figure 2a presents SEM images of the developed porous Au on flat Au IDEs at different magnifications, confirming the successful formation of highly porous Au networks using a dynamic bubbling electrodeposition technique (details in the experimental section). This well-ordered porous Au network enhances electron transport through the IDEs and supports effective PANI deposition, as confirmed by SEM images of PANI deposited on porous Au IDEs (Figure 2b). Notably, the PANI deposition (optimized with a 20 s deposition) on porous Au IDEs shows higher mass loading, with deposition current larger than when deposited under the same conditions on flat Au IDEs (Figure S2), indirectly confirming more effective PANI deposition on the porous Au networks. Exact mass measurements on IDEs are challenging due to the small device footprint (approximately 0.2 cm^2^ active electrode area); hence, mass loading is not reported. However, the morphology of PANI remains consistent, with identical nanowire-like networks and porosities, when deposited onto flat Au IDEs. Figure S3a presents SEM images of flat Au IDEs at various magnifications, while Figure S3b shows SEM images of PANI deposited on flat Au IDEs (SMSC) at different magnifications, revealing a nanowire-like network with distinct porosities. These PANI structures promote the effective diffusion of electrolyte ions, which is essential for optimal electrolyte interactions in gel electrolytes. To further investigate the quality of electrodeposited PANI, it was deposited onto Au-coated PET and titanium foil substrates under the same conditions. Figure 2c shows the XRD pattern of the deposited PANI, with distinct peaks at approximately 15.13°, 20.41°, and 25.69° corresponding to the (011), (020), and (200) reflections, respectively, indicating crystalline regions in an amorphous matrix.^16^ These peaks are attributed to the repeat unit of the PANI chain and the periodicity perpendicular and parallel to the polymer backbone chain. Similarly, the Raman spectrum of PANI (Figure 2d) shows characteristic peaks: C–H bending deformation in the benzenoid ring at 1185 cm^–1^, C–N^+^ stretching at 1343 cm^–1^, C=N stretching vibration at 1500 cm^–1^, and C=C stretching of quinoid at 1595 cm^–1^, confirming successful electrodeposition and purity of PANI.^17^

**Figure 2:**
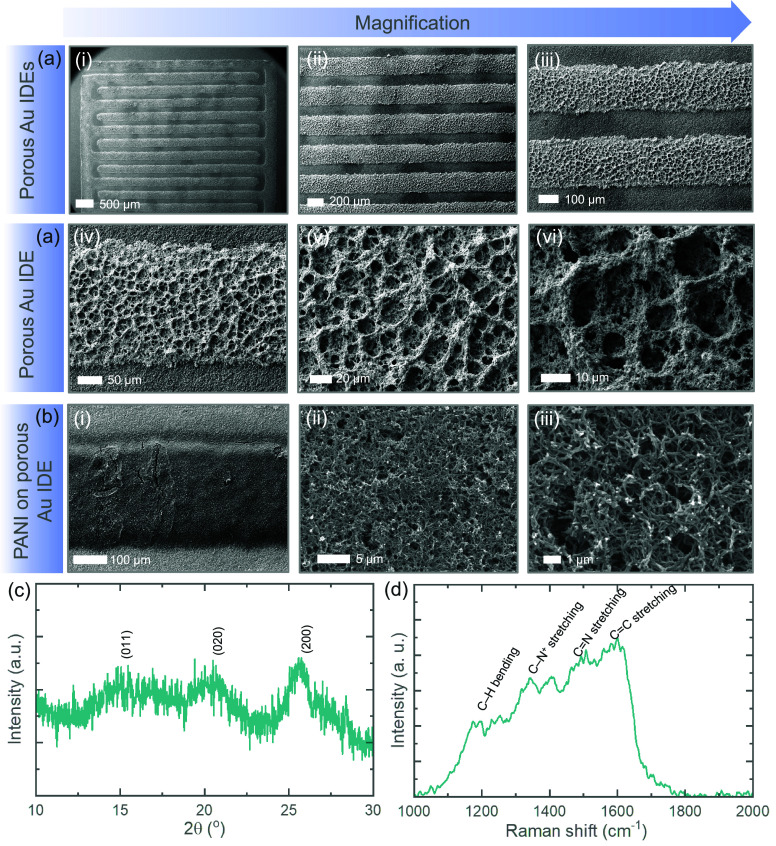
(a) SEM images of porous Au IDEs at different magnifications, demonstrating the successful development of porous Au networks on a flat Au IDE. (b) SEM images of PANI coated onto porous Au IDE at different magnifications, showing that PANI fully covers the porous Au networks after 20 s of electrodeposition. (c) XRD and (d) Raman spectra of the deposited PANI.

Next, we evaluated the electrochemical responses of SMSC and P-SMSC in a gel electrolyte consisting of H_3_PO_4_ in a PVA matrix (see the digital image of the prepared gel electrolyte in Figure S4a). For testing, we directly immersed the devices in the electrolyte cuvette and sealed them with parafilm to minimize changes in electrolyte viscosity due to water evaporation (Figure S4b). The electrochemical performance tests included cyclic voltammetry (CV) at different scan rates (10 to 100 mV/s) and galvanostatic charge–discharge (GCD) at various currents (0.2 to 2.5 mA) over a voltage window of 0.8 V. Initially, we performed 10 s PANI deposition and conducted measurements as shown in Figure S5. Comparative CVs of SMSC-10s and P-SMSC-10s with 10 s PANI deposition are shown in Figure S5c,d at scan rates of 10 and 100 mV/s, respectively. As expected, the P-SMSC-10s demonstrated significantly better charge storage performance, with enhancements in areal capacitance of ∼373% and ∼338% even with the same PANI electrodeposition time of 10 s. This improvement is mainly due to (i) Porous Au IDEs allowing more effective and higher PANI deposition under the same conditions as flat Au IDE (Figure S2), (ii) Porous Au IDEs offering rapid electron transport throughout the porous Au framework and hence lower charge transfer resistance (further supported by the Nyquist plot), and (iii) Rapid 3D diffusion of electrolyte ions (protons) for effective charge storage via pseudocapacitive mechanisms. Notably, in both devices, PANI forms nanowire-like frameworks, which further enhance charge storage in microscale electrode geometries. Additionally, we examined the CVs of porous Au IDEs and flat Au IDEs without PANI in the PVA-H_3_PO_4_ gel electrolyte (see Figure S6). The results show that the porous Au IDEs exhibit higher current compared to the flat IDEs, which further contributes to the enhanced overall charge storage response in the P-SMSC. All these characteristics of more samples deposited with PANI for extended time (20 s) on porous Au IDEs contribute to improve charge storage performance. To further increase loading of PANI, we extended the deposition time to 20 s, optimizing charge storage through the deposition parameters. The 20 s deposition provided optimal charge-storage performance, and these devices were considered throughout the manuscript unless otherwise noted. Figure S7a,b shows the CVs of SMSC and P-SMSC with PANI deposited for 20 s, demonstrating a stable and reversible charge storage from low (10 mV/s) to high (100 mV/s) scan rates. As expected, P-SMSC exhibited superior charge storage performance compared to SMSC. At scan rates of 10 mV/s (Figure S7c) and 100 mV/s (Figure S7d), the areal capacitance enhancements were ∼201% and ∼192%, respectively. Remarkably, even with just 10 s of PANI deposition on porous Au IDEs (P-SMSC-10s), the areal capacitance surpassed that of 20 s of PANI deposition on flat Au IDEs (SMSC). The comparative areal capacitance versus scan rate plot (Figure S7e) clearly shows much higher areal capacitances of PANI deposition on porous Au IDEs even from lower to higher scans range. For instance, at 10 mV/s, the measured areal capacitances are 17 mF/cm^2^, 81 mF/cm^2^, 53 mF/cm^2^, and 160 mF/cm^2^ for SMSC-10 s, P-SMSC-10 s, SMSC, and P-SMSC, respectively, and 14 mF/cm^2^, 61 mF/cm^2^, 44 mF/cm^2^, and 127 mF/cm^2^ at a scan rate of 100 mV/s. The overall charge storage performance of PANI in the PVA/H_3_PO_4_ electrolyte results from a combination of electric double-layer capacitance (EDLC), arising from ion adsorption and desorption at the electrode/electrolyte interface, and pseudocapacitance, which is due to the rapid and reversible redox reactions within the PANI structure. This dual mechanism allows PANI-based electrodes to attain high capacitance, significant energy density, and excellent cycling stability (see further).

To gain a deeper understanding of the charge storage performance of the devices, we extended the electrochemical assessment to GCD tests, conducted at various currents (Figure 3a,b) within the same voltage range from 0 to 0.8 V used in the CV tests. Consistent with the CV profiles, the comparative GCDs of SMSC and P-SMSC tested at 0.2 mA (Figure 3c) revealed superior charge storage performance in the P-SMSC configuration compared to SMSC, with measured areal capacitances of 23 mF/cm^2^ and 60 mF/cm^2^ (∼160% enhancement). Similarly, even at a higher areal current of 2.5 mA, the P-SMSC demonstrates 19.5 mF/cm^2^ while SMSC shows 56 mF/cm^2^ (∼187% enhancement) for 20 s PANI deposition. Additionally, GCDs of the SMSC-10 s and P-SMSC-10 s are provided in the Supporting Information (Figure S8), further confirming the better charge storage performance of P-SMSC compared to SMSC during 10 s PANI deposition. Detailed areal capacitance comparisons of the devices can be found in the rate test plots (Figure 3e). Impressively, there is no significant change in the areal capacitance of the P-SMSC even as the currents increase from 0.2 to 2.5 mA, significantly higher than those of the SMSC devices. Consistent with the CV results, the 10-s PANI deposition in P-SMSC demonstrates higher capacitance than the 20-s PANI deposition in SMSC. This highlights that designing porous microelectrodes as current collectors significantly influences the overall performance of microscale energy storage devices within the limited active area. Hence, smart electrode design is crucial for boosting charge storage performance within a limited device footprint.

**Figure 3:**
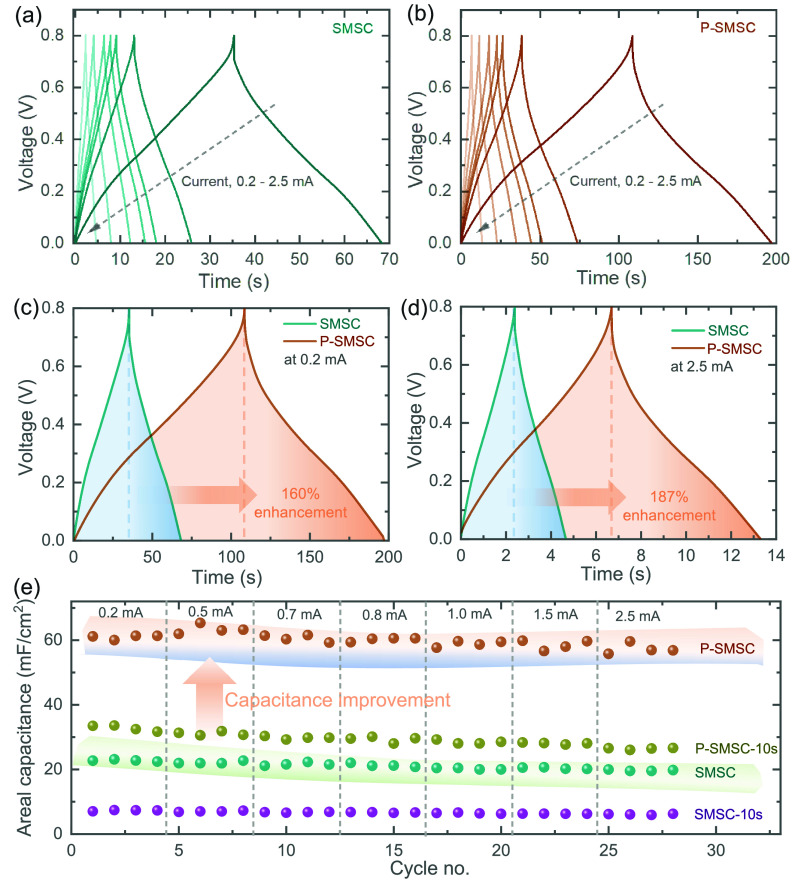
GCDs of the (a) SMSC and (b) P-SMSC at different currents of 0.2, 0.5, 0.7, 0.8, 1.0, 1.5, and 2.5 mA over the voltage window of 0–0.8 V. Comparative GCDs of the SMSC and P-SMSC at areal currents of (c) 0.2 mA and (d) 2.5 mA. (e) Comparative rate tests of the SMSC-10 s, P-SMSC-10 s, SMSC, and P-SMSC devices, demonstrating higher and stable areal capacitances of P-SMSC even at different currents from 0.2 mA to 2.5 mA.

Next, we extended the Electrochemical Impedance Spectroscopy tests of SMSC and P-SMSC with 20 s PANI deposition to understand the charge transfer kinetics of the devices. As shown in Figure 4a, the Nyquist plot confirms that P-SMSC not only improves charge storage performance but also enhances charge transfer kinetics. The measured equivalent series resistance decreases from 4.0 Ω in SMSC to 3.6 Ω in P-SMSC. Figure 4b shows the Bode plots, depicting the impedance phase angle with respect to the frequency of SMSC and P-SMSC. The measured high characteristic frequencies are 4400 and 327 Hz, corresponding to time constants of 0.23 and 3 ms at a phase angle of −45°. The relatively higher characteristic frequencies suggest that porous Au IDE-based devices can have AC line filtering capability. Likewise, as expected, P-SMSC-10s demonstrate lower equivalent series resistance than SMSC-10s (see Figure S8e). To understand the long-term capacitance stability, we extended the cycling GCD performance to 1000 cycles at 0.27 mA after stabilizing the capacitances of the devices, as shown in Figure 4c. It is noted that the capacitance retentions are measured to be 80% and 81% for SMSC and P-SMSC, respectively. These results demonstrate that both SMSC and P-SMSC devices display similar trends, with a gradual decrease in capacitance. This decrease is mainly due to the stability of PANI materials in the PVA-H_3_PO_4_ electrolyte rather than the electrode geometries. The digital images of the SMSC and P-SMSC before and after cycling reveal that even after 1000 cycles, the electrode materials remain adhered to the porous Au microelectrodes without any peeling issues in the inset images (Figure 4c). This is noteworthy for microscale energy storage devices and can be attributed to the effective adhesion of PANI to the porous Au IDEs, as well as the mechanical support provided by the PVA-H_3_PO_4_ gel electrolyte, which helps prevent peeling. This confirms that the P-SMSC not only offers higher charge storage performance but also maintains stability over extended cycles. To explore the morphologies and composition of the electrodes after cycling tests, we performed SEM imaging (Figure 4d) and Raman (Figure S9) characterization of the cycled electrode materials in SMSC and P-SMSC. Completely removing the gel electrolyte from the electrode surfaces is challenging, but we managed to clean them sufficiently for characterization. Figure 4e shows the SEM image of the cycled SMSC. Interestingly, there is no significant change in the morphology of the PANI nanowire-like framework even after 1000 cycles, maintaining the identical morphology of the materials, which is an additional advantage of our microdevices. Similarly, a consistent observation is noted in cycled P-SMSC (Figure 4e). The SEM images at various magnifications clearly reveal and support the morphological and structural stabilities of the microelectrodes. Similarly, Figure S9 shows the Raman spectra after cycling. The characteristic peaks of C–H bending deformation in the benzenoid ring (at 1190 cm^–1^), C–N^+^ stretching (at 1365 cm^–1^), and C=C stretching of quinoid (at 1600 cm^–1^) related to the PANI are still detectable on the PANI electrodes. However, maintaining the identical composition of PANI is expected because of the rapid and efficient interaction of protons with the surface of PANI for pseudocapacitive response, as well as electrical double layer capacitance without destroying the electrode composition and morphology.

**Figure 4:**
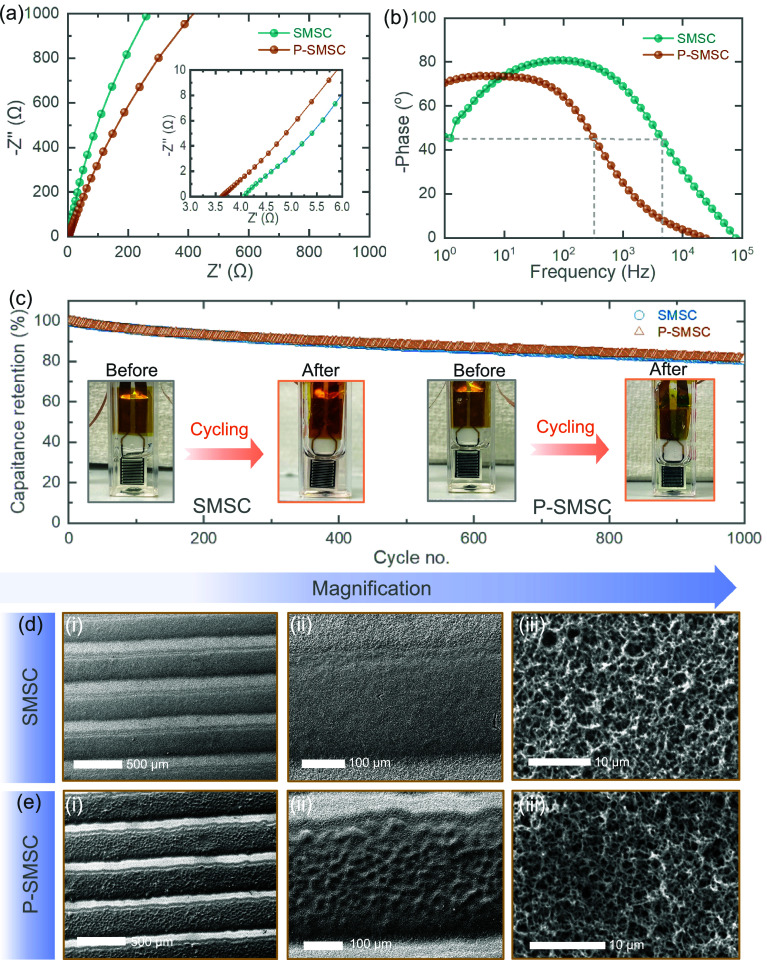
(a) Nyquist plot of the SMSC and P-SMSC, showing lower charge transfer resistance in the P-SMSC compared to the SMSC with 20 s PANI deposition. (b) Bode plots of SMSC and P-SMSC. (c) Long-term cycling stability test of SMSC and P-SMSC at 0.27 mA for 1000 cycles. Insets are the digital images of SMSC and P-SMSC before and after cycling, showing no peeling of materials from the flat Au IDEs add porous Au IDEs. (d, e) Post-mortem SEM images of cycled SMSC and P-SMSC at different magnifications, revealing PANI nanowire-like matrix similar to the initial electrodes.

Additionally, we computed the areal energies and areal powers of SMSC-10s, P-SMSC-10s, SMSC, and P-SMSC devices at different currents ranging from 0.2 to 2.5 mA, as shown in Figure S10. The results indicate that P-SMSC demonstrates substantially higher areal energies compared to SMSC, SMSC-10s, and P-SMSC-10s, with P-SMSC-10s showing the second highest values; these findings align with the CVs and GCDs results. For example, the calculated areal energies at 0.2 mA are 0.62 μWh/cm^2^, 2.97 μWh/cm^2^, 2.02 μWh/cm^2^, and 5.44 μWh/cm^2^ for SMSC-10s, P-SMSC-10s, SMSC, and P-SMSC, respectively, and at 2.5 mA, they are 0.54 μWh/cm^2^, 2.36 μWh/cm^2^, 1.77 μWh/cm^2^, and 4.97 μWh/cm^2^. These values for P-SMSC surpass those of most previously reported high-performance micro-supercapacitors, including both symmetric and asymmetric configurations. The Ragone plot in Figure 5 and Table S1 clearly demonstrate the superior performance of our P-SMSC, suggesting that it offers outstanding charge storage capabilities, outperforming most reported micro-supercapacitors. Our advanced electrode design approach not only provides higher charge storage but also ensures stable performance. Furthermore, Figure S11 illustrates the volumetric energy at different volumetric power levels, showing that the volumetric energies of P-SMSC are (∼3.5 mWh/cm^3^) to those of SMSC (∼5 mWh/cm^3^) with a 20 s PANI deposition. Although gold is a precious metal that affects material costs, it is widely used in electronic devices. However, the processing method detailed in this work is both efficient and rapid, providing precise control over porosity without the need for specialized cleanroom facilities usually required for micro- and nanomaterial processing. Additionally, the fabrication technique for porous Au described in the manuscript is readily scalable to wafer-sized applications with minimal difficulties.

**Figure 5:**
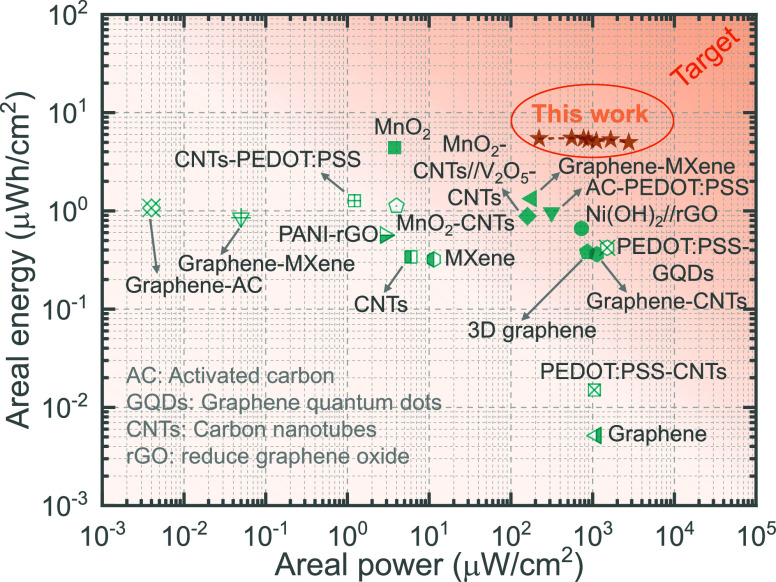
*Ragone plot offering a comparative overview of our P-SMSC with previously reported micro-supercapacitors, encompassing both symmetric and asymmetric designs: graphene*,^18^*PEDOT:PSS-CNTs*,^19^*3D graphene*,^20^*MXene*,^21^*CNTs*,^22^*Graphene-CNTs*,^23^*MnO*_*2*_*–CNTs//V*_*2*_*O*_*5*_*–CNTs*,^24^*Ni(OH)*_*2*_*//rGO*,^25^*AC-PEDOT:PSS*,^26^*Graphene-MXene*,^27,28^*CNTs-PEDOT:PSS*,^29^*PEDOT:PSS-GQDs*,^30^*PANI-rGO*,^31^*Graphene-AC*,^32^*MnO_2_*,^33^*MnO_2_–CNTs*.^34^

In summary, this study focuses on developing porous microelectrodes for high-performance on-chip planar micro-supercapacitors by using porous Au IDEs for effective PANI deposition as active materials. These proposed porous Au IDEs-based symmetric micro-supercapacitors exhibit a remarkable 187% increase in areal capacitance at 2.5 mA compared to their conventional flat Au IDE counterparts, despite using the same amount of active material. As a result, our P-SMSCs achieve an areal capacitance of 60 mF/cm^2^, a peak areal energy of 5.44 μWh/cm^2^, and an areal power of 2778 μW/cm^2^, surpassing most reported micro-supercapacitors to date. Our P-SMSCs also demonstrate capacitance stability with 81% retention after 1000 cycles and maintain the same states and morphologies of the electrodes, supporting the stability of our approach. This research paves the way for exploring advanced porous current collectors to significantly enhance the charge storage performance of micro-supercapacitors within a limited device footprint. This method allows for the precise loading of active materials onto confined porous microelectrodes, thereby achieving high-performance planar microscale energy storage devices.

## Supporting Information Available

Experimental Section; 2D and 3D profilometer maps (**Figure S1**); Current profiles of PANI deposition (**Figure S2**); SEM images of flat Au IDEs and SMSC (**Figure S3**); Digital images of PVA-H_3_PO_4_ gel electrolyte and a device (**Figure S4**); CVs of SMSC-10s and P-SMSC-10s (**Figure S5**); CVs of flat Au and porous Au IDEs (**Figure S6**); CVs of SMSC and P-SMSC and comparative areal capacitance (**Figure S7**); GCDs and EIS of SMSC-10s and P-SMSC-10s (**Figure S8**); Raman spectrum of cycled PANI electrode (**Figure S9**); Areal energies at various areal powers of the devices (**Figure S10**) (PDF)
