# Supplementary material for: Advanced Porous Gold-PANI Micro-Electrodes for High-Performance On-Chip Micro-Supercapacitors
Source: Nano Lett. 2024 Aug 26;24(35):11059–66. doi: 10.1021/acs.nanolett.4c03194 (PMC11378337; doi:10.1021/acs.nanolett.4c03194)
Supplement: Supplementary file 1 — nl4c03194_si_001.pdf [file nl4c03194_si_001.pdf]

Supporting Information
for
Advanced Porous Gold-PANI Micro-Electrodes for High-Performance On-Chip Micro-Supercapacitors

Nibagani Naresh,[1] Yijia Zhu,[1] Yujia Fan,[1] Jingli Luo,[1] Tianlei Wang,[2] Ivan P. Parkin,[2] Buddha Deka Boruah[1,*]

[1]Institute for Materials Discovery, University College London, London WC1E 7JE, United Kingdom

[2]Department of Chemistry, University College London, London, WC1H 0AJ, UK

Corresponding author: Dr. Buddha Deka Boruah

Email: b.boruah@ucl.ac.uk

Experimental Sections:

Materials: Gold (III) chloride trihydrate ($AuCl_4H_7O_3$) and ammonium chloride (NH_4Cl), supplied by Sigma-Aldrich, were used for the fabrication of porous gold. Sulfuric acid (H_2SO_4) and aniline, also from Sigma-Aldrich, were utilized in the electrodeposition of PANI. For the preparation of the gel electrolyte, poly(vinyl alcohol) and 85 wt% phosphoric acid (H_3PO_4) were procured from Sigma-Aldrich as well.

Electrodeposition of porous Au: The flat Au IDEs (Interdigitated Gold Electrodes with 200-micron lines and gaps, DRP-IDEAU200) were received from Metrohm U.K. Ltd. Before use, the received flat Au IDEs were cleaned with isopropanol and distilled water, then dried with nitrogen gas. To prepare the deposition solution, a mixture of 0.1 M gold (III) chloride trihydrate ($AuCl_4H_7O_3$) and 2 M ammonium chloride (NH_4Cl) was dissolved in 20 ml of distilled water. This solution was then used to electrodeposit porous Au on the flat IDEs device using the dynamic bubble method[1]. To ensure consistency, the solution was stirred continuously for one hour at room temperature. Pt wire and Ag/AgCl were used as the counter and reference electrodes, respectively. The electrodeposition of porous Au onto the flat IDE devices was performed at a voltage of -2 V for 5 seconds. After electrodeposition, the devices were washed with distilled water and allowed to dry before use. An Autolab electrochemical workstation was employed to carry out the electrodeposition process.

Electrodeposition of PANI: PANI was electrodeposited on both porous Au IDEs and flat Au IDEs devices using an electrodeposition technique. The PANI electrolyte solution was prepared by mixing 1M H_2SO_4 (95–97%) and 0.5 M aniline in 50 ml of distilled water, and the solution was continuously stirred on a magnetic stirrer for 3 hours to achieve homogeneity. Pt wire and Ag/AgCl were used as the counter and reference electrodes, respectively. The homogeneous PANI solution was electrodeposited on both types of electrodes at a potential of 0.85 V for 10 and 20 seconds. After deposition, the electrodes were washed with distilled water and allowed to dry.

Material characterizations: X-ray diffraction (XRD) measurement of PANI was carried out using (AERIS PANalytical Research Edition) using Cu K± radiation (λ = 0.154 nm) at 40 kV and 7.5 mA from 10 to 80°, scanning at 5° per minute. Raman spectroscopy analysis of PANI was measured using Renishaw inVia™ confocal Raman Microscope using a 532 nm

wavelength laser from 100 to 3200 cm^{-1}. Scanning electron microscope (SEM) (Zeiss EVO LS15) was used to investigate the morphology of the devices. Further, the surface profile of the devices was investigated using a stylus profilometer (Bruker DektakXT).

Gel electrolyte: The H_3PO_4-PVA gel electrolyte is prepared by mixing polyvinyl alcohol (PVA) (3 g) with 30 mL of DI water while being stirred at 95 °C for one hour. After dissolving PVA completely, 6 ml of H_3PO_4 was added to the solution and stirred continuously for a few hours to obtain H_3PO_4 gel electrolyte.

Electrochemical Test: To test the developed micro-supercapacitors, the two ends of the electrodeposited IDEs device were fixed with copper foil using silver paste. The devices were then immersed vertically in an electrolyte-filled cuvette (Fisherbrand Disposable Cuvettes 14955125), and the top of the cuvette was sealed with parafilm. The assembled devices were connected to an electrochemical tester (Biologic) for cyclic voltammetry (CV) at scan rates ranging from 10 to 100 mV/s over a 0.8 V voltage window and for galvanostatic charge-discharge (GCD) measurements at currents from 0.2 to 2.5 mA. Long-term cycle measurements were conducted using a Neware battery tester. Electrochemical impedance spectroscopy (EIS) measurements were performed using an Autolab electrochemical workstation, with frequency testing ranging from 10 mHz to 100 kHz at 10 mV voltage amplitude.

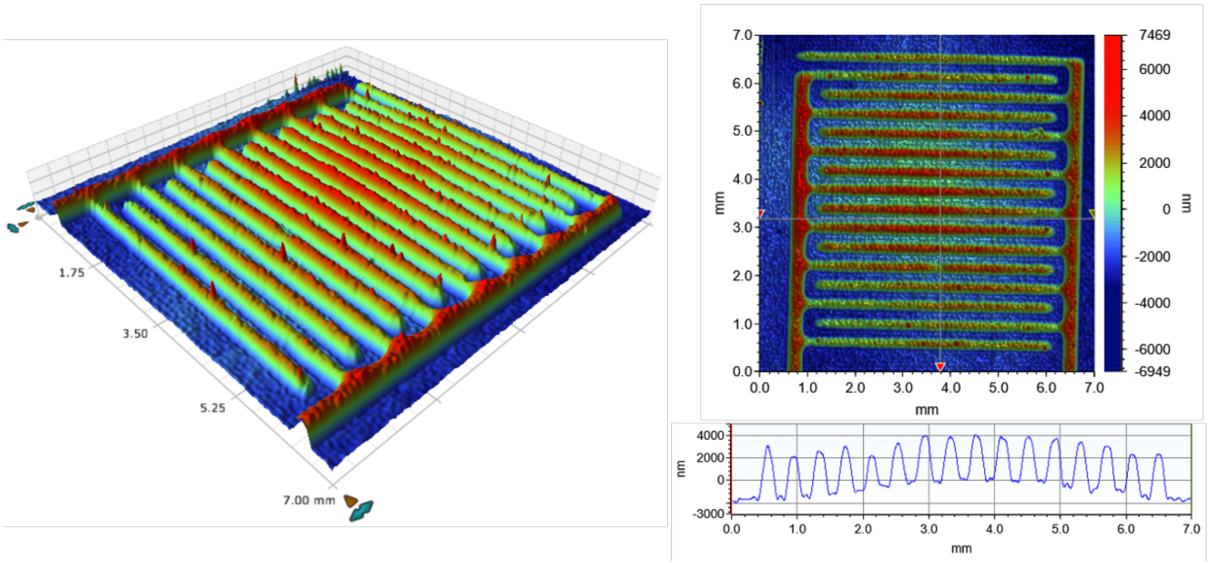

Figure S1. *2D and 3D profilometer maps of the flat Au IDEs, showing a measured thickness of 4 µm.*

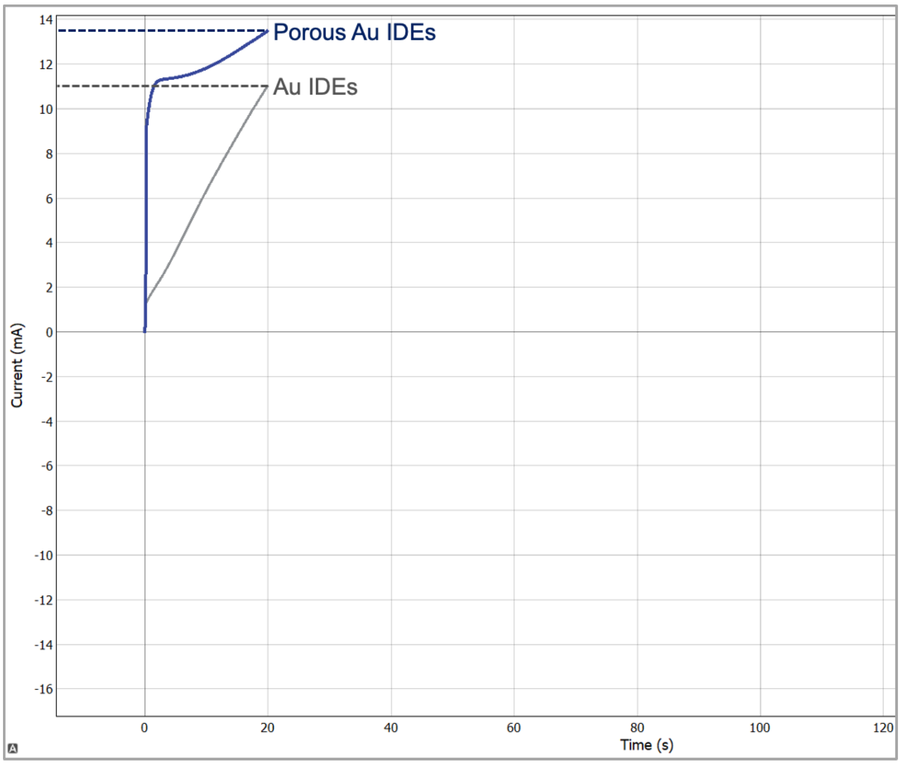

Figure S2. *Current profiles of PANI deposition on both flat Au IDEs and porous Au IDEs.*

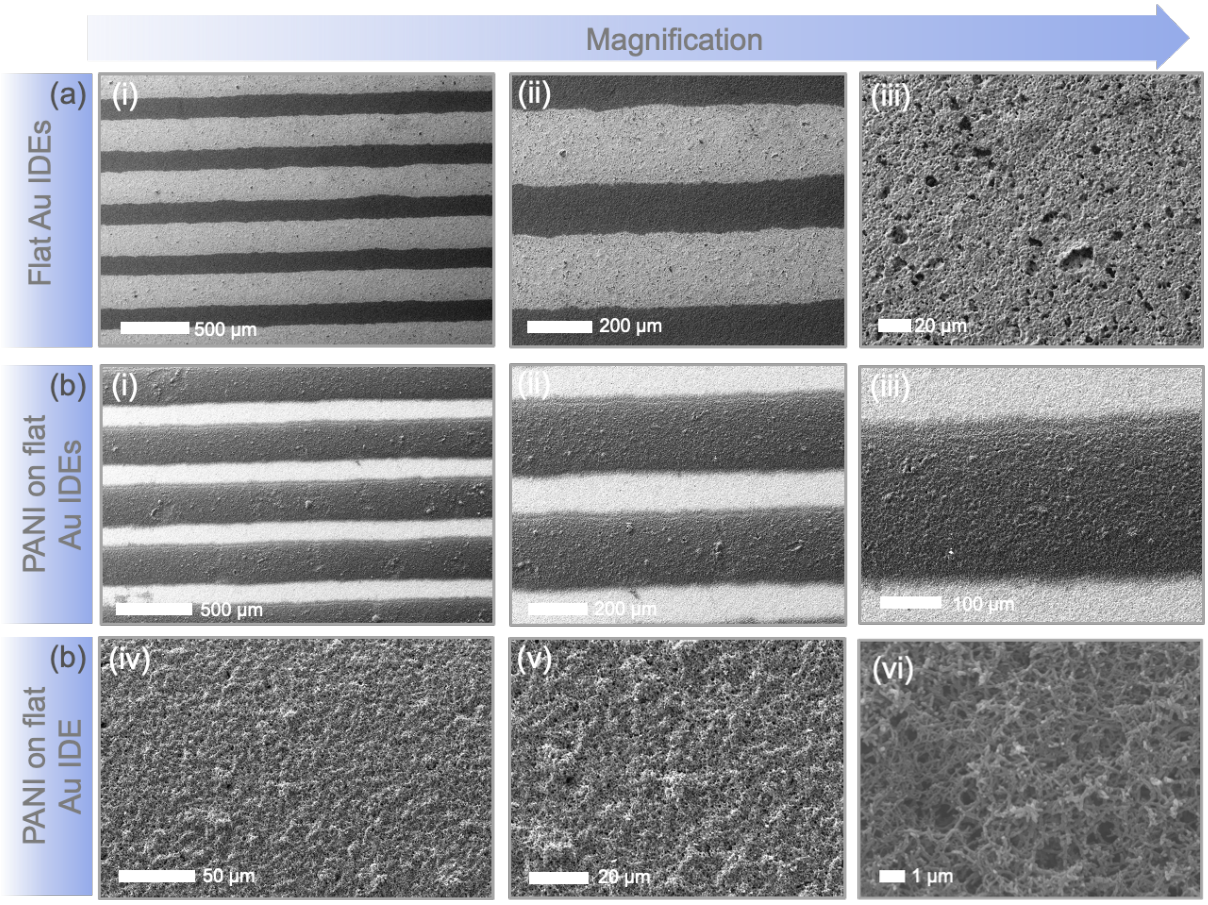

Figure S3. *(a) SEM images of flat Au IDEs at various magnifications. (b) SEM images of PANI deposited on flat Au IDEs at different magnifications after 20 seconds of deposition, highlighting the porous nanowire-like network of the deposited PANI.*

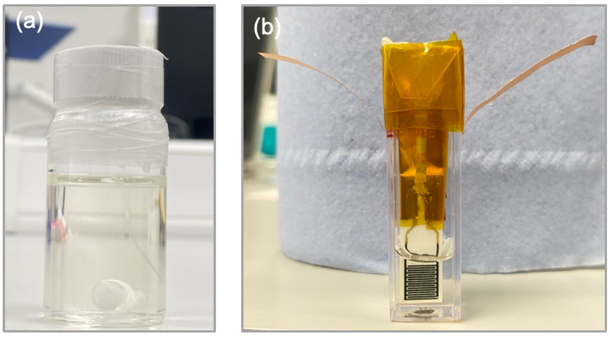

Figure S4. *Digital images of (a) the as-prepared PVA-H_3PO_4 gel electrolyte and (b) a device immersed in the gel electrolyte for testing.*

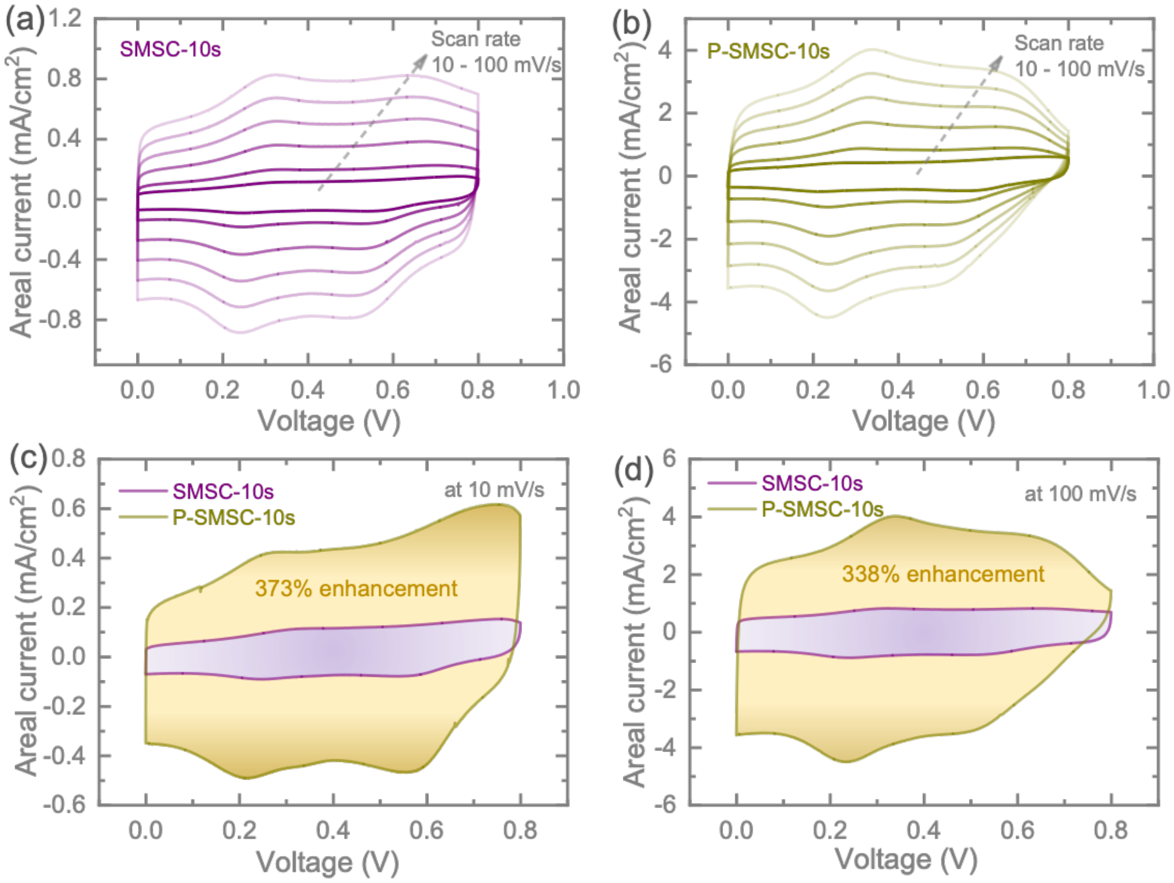

Figure S5. *CVs of (a) SMSC-10s and (b) P-SMSC-10s at various scan rates. Comparative CVs of SMSC-10s and P-SMSC-10s at scan rates of (c) 10 mV/s and (d) 100 mV/s, showing significant enhancements in charge storage performance for P-SMSC-10s, with increases of 373% at 10 mV/s and 338% at 100 mV/s compared to SMSC-10s.*

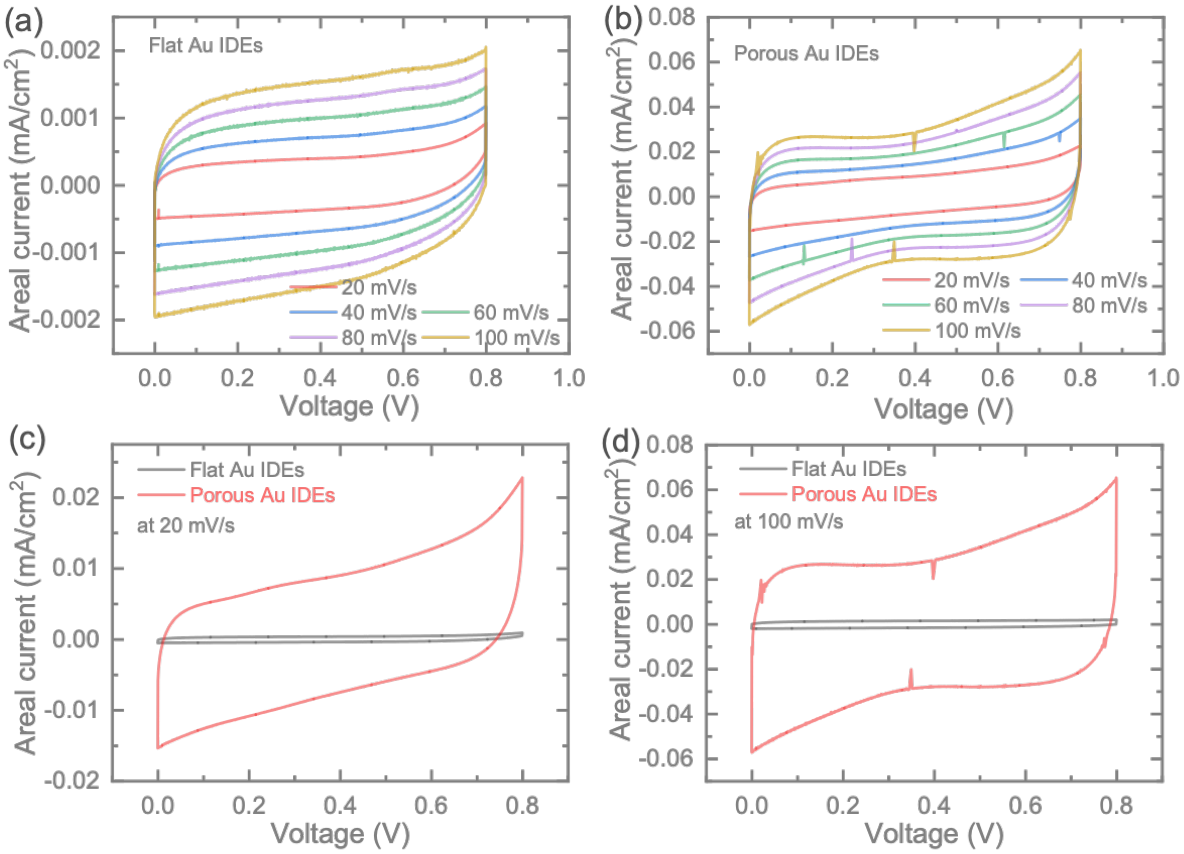

Figure S6. *CVs of (a) flat Au IDEs and (b) porous Au IDEs at various scan rates. Comparative CVs of flat Au IDEs and porous Au IDEs at scan rates of (c) 20 mV/s and (d) 100 mV/s.*

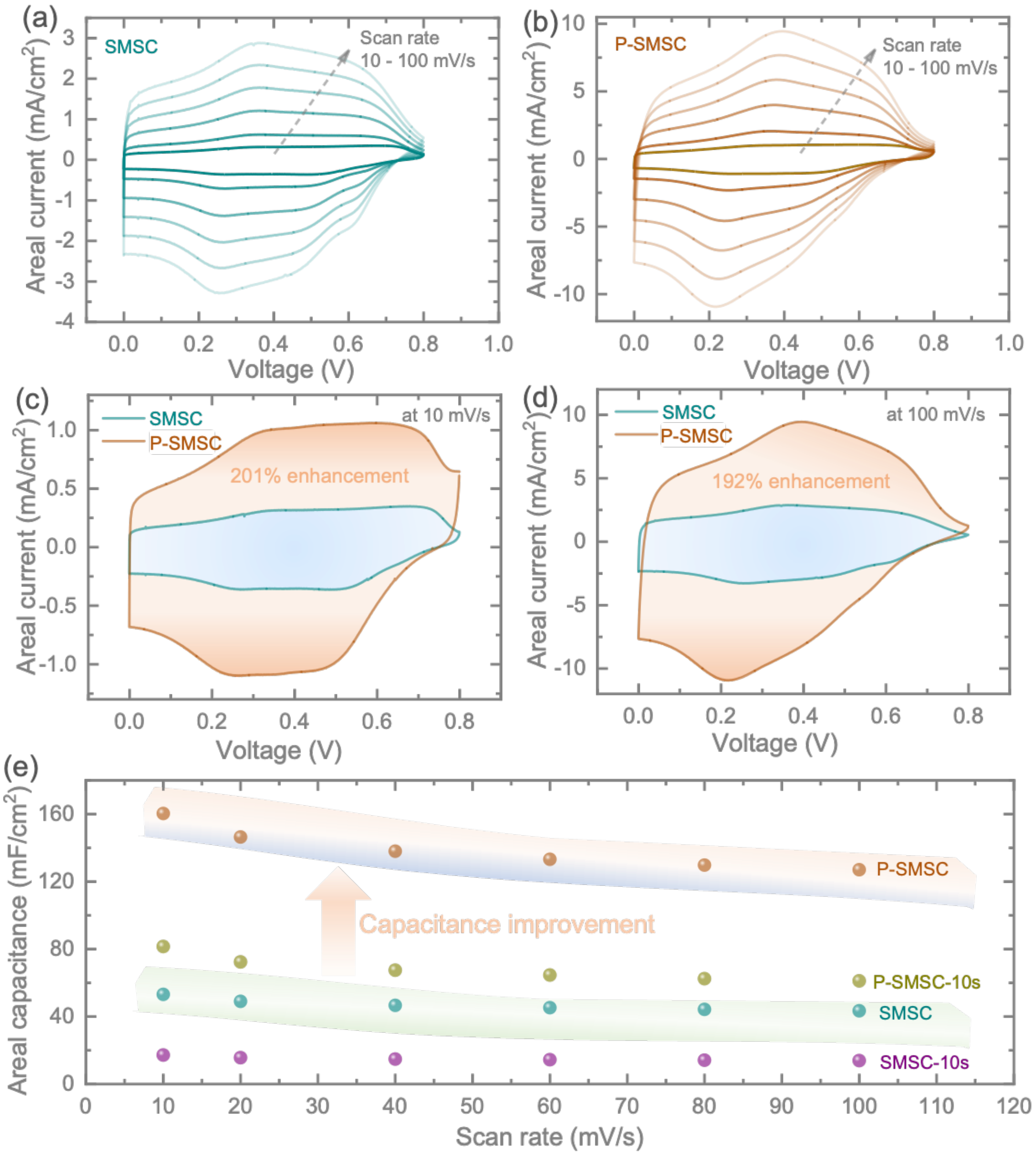

Figure S7. *CVs of (a) SMSC and (b) P-SMSC at different scan rates ranging from 10 to 100 mV/s for 20 seconds of PANI deposition. Comparative CVs of SMSC and P-SMSC after 20 seconds of PANI deposition at scan rates of (c) 10 mV/s and (d) 100 mV/s. (e) Comparative areal capacitance of SMSC and P-SMSC deposited for 10 and 20 seconds at various scan rates, highlighting the significantly higher areal capacitance response of P-SMSCs compared to SMSCs. Devices with 10-second and 20-second PANI deposition are referred to as SMSC-10 s, P-SMSC-10 s, SMSC, and P-SMSC, respectively in this plot.*

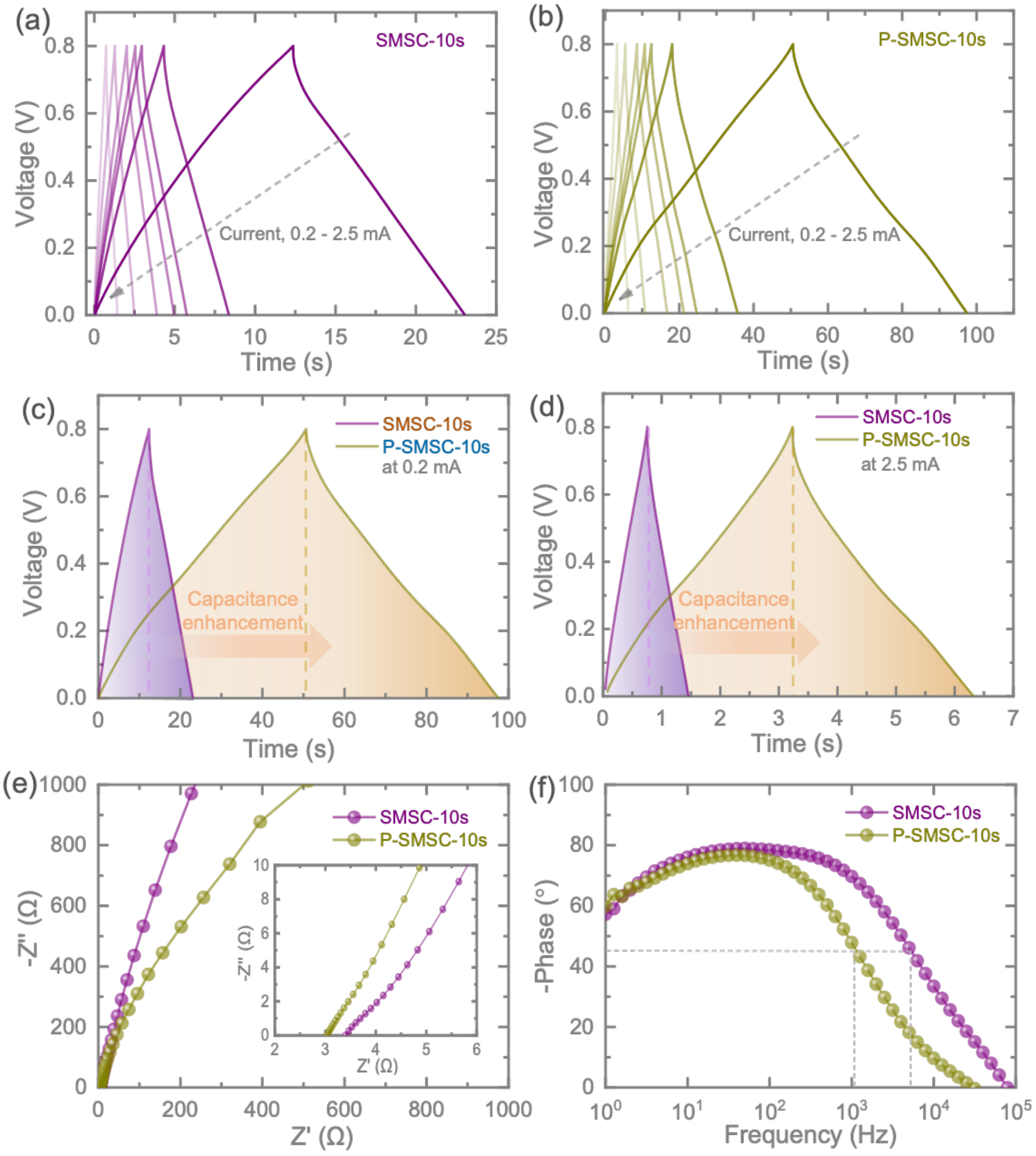

Figure S8. *GCDs of (a) SMSC-10s and (b) P-SMSC-10s at various currents. Comparative GCDs of SMSC-10s and P-SMSC-10s at currents of (c) 0.2 mA and (d) 2.5 mA, demonstrating significant enhancements in charge storage performance for P-SMSC-10s compared to SMSC-10s. (e) Nyquist and (f) Bode plots of the SMSC-10s and P-SMSC-10s devices.*

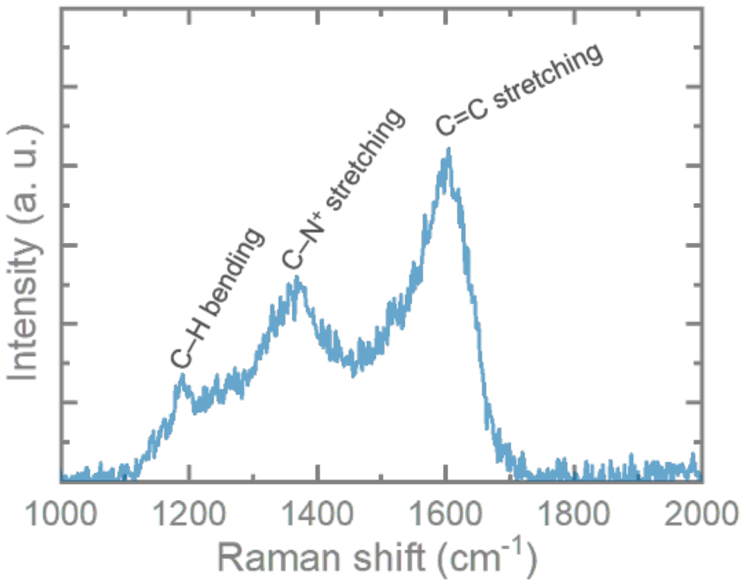

Figure S9. *Raman spectrum of cycled PANI electrode.*

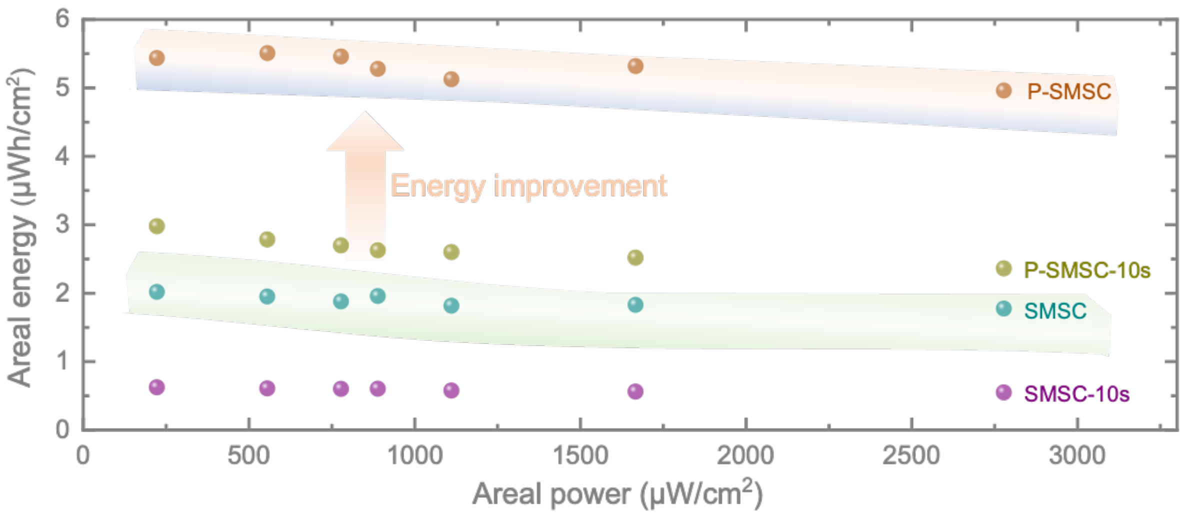

Figure S10. *Areal energies at various areal powers of the devices, demonstrating the superior charge storage performance of P-SMSC compared to other devices, including SMSC, P-SMSC-10s, and SMSC-10s. Notably, even a 10-second deposition of PANI on porous Au IDEs (P-SMSC-10s) results in better charge storage performance than a 20-second PANI deposition on flat Au IDEs.*

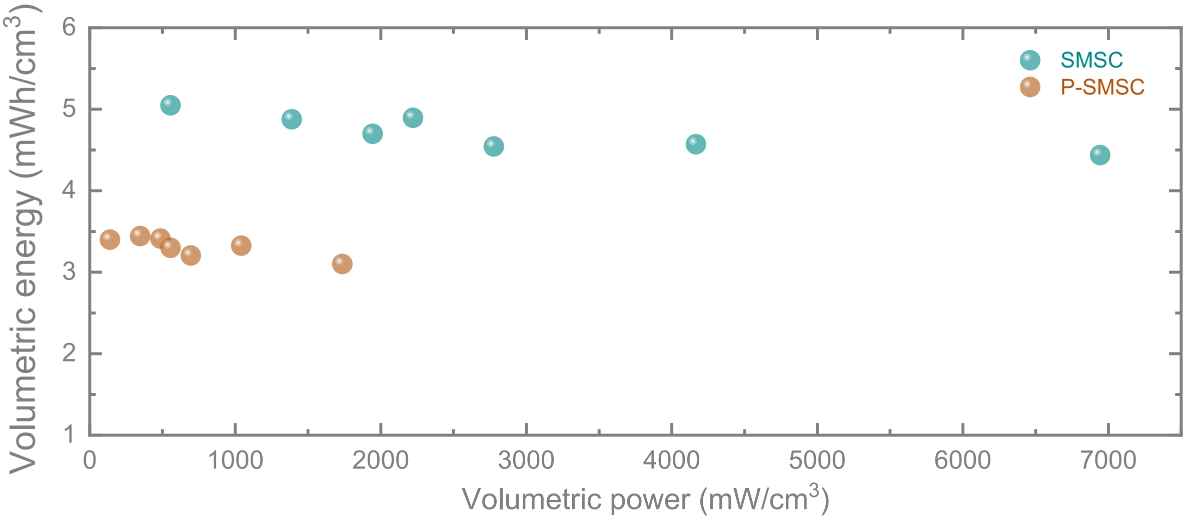

Figure S11. *Volumetric energies at various volumetric powers of the P-SMSC and SMSC devices for 20-second PANI deposition.*

S.I. No	Sample Name	Areal Energy (µWh/cm²)	Areal Power (µW/cm²)	Reference number
**1**	***P-SMSC***	**5.44**	**2778**	**This work**
2	CNTs-PDOT:PSS	1.27	1.22	19
3	PANI-rGO	0.564	2.898	31
4	Graphene-Mxene	1.33	180	27
5	Graphene-AC	1.07	0.004	32
6	CNTs	0.34	6	22
7	MnO_2	4.4	3.8	33
8	MnO_2-CNTs/V_2O_5-CNTs	0.88	160	24
9	MnO_2-CNTs	1.12	3.99	34
10	Mxene	11.4	0.32	21
11	3D Graphene	0.38	860	20
12	PEDOT:PSS-CNTs	0.015	1050	29
13	Graphene	0.00516	1112	18
14	Graphene-Mxene	0.85	0.05	27
15	AC-PEDOT:PSS	2.79	800	26
16	$Ni(OH)_2$//rGO	0.66	730	25
17	PEDOT:PSS-GQD	0.42	1510	30
18	Graphene-CNTs	0.361	1130	23

Table S1. *Comparison of areal energy and areal power of P-SMSC with data from the literature. Note: The reference numbers in the table correspond to those cited in the main paper.*

Calculations

The areal capacitance for CVs, C_s (mF/cm^2) of the devices is calculated using the formula

$$C_s = \frac{1}{s \times \Delta V \times \nu} \int_{V1}^{V2} I(V)\, dV$$

Where, s is device area in cm^2 which is 0.36 cm^2, ν is scan rate (V/s), $\Delta V = V2 - V1$ is the voltage window which is 0.8 V.

The areal capacitance from GCDs, C_s (mF/cm^2) of the devices is calculated using the formula

$$C_s = \frac{I \times t_d}{s \times \Delta V}$$

Where, t_d is the discharge time in second and I applied current in mA.

The areal energy, E_s ($\mu Wh/cm^2$) and areal power, P_s ($\mu W/cm^2$) of the devices are computed using the following relationships:

$$E_s = \frac{1}{2} \times C_s \times \frac{\Delta V^2}{3600}$$

and

$$P_s = \frac{E_s}{t_d} \times 3600$$

References:

(1) Cherevko, S.; Chung, C. H. Direct Electrodeposition of Nanoporous Gold with Controlled Multimodal Pore Size Distribution. *Electrochem. commun.* **2011**, *13* (1), 16–19.
